# Additive screening and formula optimization of microbial inhibitor having disease prevention and growth promotion effects on *Avena sativa*

**DOI:** 10.3389/fmicb.2023.1208591

**Published:** 2023-07-20

**Authors:** Jiangui Zhang, Tuo Yao, Wenlong Gong, Yamin Gao, Guiqin Zhao

**Affiliations:** ^1^College of Grassland Science, Gansu Agricultural University, Lanzhou, Gansu, China; ^2^Key Laboratory of Grassland Ecosystem of Ministry of Education, Lanzhou, Gansu, China

**Keywords:** mix biocontrol bacteria, additive screening, formulation optimization, *Avena sativa*, disease control, growth promotion

## Abstract

In order to develop environment friendly microbial inhibitor that can also control disease and promote oat (*Avena sativa*) growth, the growth rate method and response surface methodology were used to screen wetting agents, preservatives and protective agents at optimal concentrations in this study. Antagonistic activity of the tested bacterium and cell-free fermentation liquid against pathogenic fungi was evaluated on potato dextrose agar (PDA) substratum plates by dual culture technique. Oxford cup method was used to measure antagonistic reaction between screened bacteria. According to each screened bacteria with 50 mL were mixed and cultured in Luria-bertani (LB) substratum. Additives of Wetting agents, UV-protectors, and preservatives were screened by single factor test on the growth concentration of screened mixed bacteria. Afterwards, the optimal additives and concentrations were screened by Box-Behnken method. The microbial inhibitor was detected according to national standards GB20287-2006 and tested on oat in a pot experiment. The results showed that: (1) Functional bacteria which including *Bacillus velezensis* and *Brevundimonas faecalis* had control effects of 50.00% to 83.29% on three pathogenic fungi, and their cell free-fermentation liquid could inhibit the growth of pathogenic fungi from 23.51% to 39.90%; (2) Tween-80 was most suitable as wetting agents for Mix biocontrol bacteria (MBB) with 1.00% mass fraction; Sorbitol was selected as UV protective agents for MBB with 0.50% mass fraction. And methyl paraben was used as a preservative for MBB, with 0.50% mass fraction; (3) The most effective adjuvant contained 14.96 mL/L Tween-80, 5.12 g/L methylparaben and 5.6 g/L sorbitol; and (4) The microbial inhibitor controlled 45.57% of oat root rot and increased plant height, root length and seedling biomass. This study provides a suitable environment for the protection of mixed biocontrol bacteria, and lays a foundation for the prevention and control of oat diseases, the promotion of growth and the improvement of quality.

## 1. Introduction

Food security has always been a topic of concern. Unfortunately, in the past few years, pesticide pollution in the air, water, and soil and deaths caused by pesticides have been serious in various countries (Karunarathne et al., 2020). Pesticide poisoning often happens when chemical pesticides are used to control a pest, and it affects humans, wildlife, plants, and beneficial insects (Wang et al., 2022). Therefore, a biological control strategy is an important alternative for this type of agriculture, and *Trichoderma* spp. and *Bacillus* spp. are well known as biological agents and have been applied to control root rot disease in many crops (Alamri et al., 2019). A microbial inhibitor has already applied to control root rot by seed dressing and plant spraying and to control plant diseases and plant growth promotion (O'Callaghan, 2016; Abbas et al., 2019). The problem is that most microbial agents are susceptible to ultraviolet, temperature, and sunlight (Compant et al., 2005; Kaewkham et al., 2016). The number of effective microorganisms in microbial inhibitors decreases and reduces their colonization on the crop root surface (Arora and Mishra, 2016). The reason for this problem is that, on the one hand, the biocontrol effect of the selected bacteria itself is not very high, but on the other hand, the protective effect of the adjuvant is insufficient. Therefore, it is imperative to screen bacteria with good biocontrol effect, and then through good additives, to ensure their role.

As the main components of biocontrol agents, microbial agents play important roles in the biological control of diseases. Common biocontrol bacteria include *Bacillus subtilis*, *Bacillus velezensis*, *Bacillus thuringiensis*, *Pseudomonas fluorescens*, *Trichoderma harzianum*, *Alternaria* spp., and *Streptomyces* (Duan et al., 2020; Müller and Behrendt, 2021). Microbial agents were found to have controlled 80% of root rot in ginseng in Jilin Province (Zhang et al., 2019). The application of microbial agents increases wheat yield by 7.7–24.2% (Chang et al., 2017). In Sweden, microbial agents increased the yield of wheat suffering from root rot by 26.37% (Al-Sadi, 2021). Additives can effectively protect the activities of microorganisms and improve the durability of microbial agents and even improve disease control effects (Yardin et al., 2000; Raymaekers et al., 2020). The use of a combination of lignosulfonate and polyethylene glycol additives in the formulation increased the survival of *Lysobacter capsici* cells living on grapevine leaves under field conditions by 10 times and caused a reduction of 71% in *Plasmopara viticola* attacks (Segarra et al., 2015). Common forms of additives include granules, suspension agents, water agents, and wettable powders (Shi et al., 2008; Yang et al., 2017). Additives commonly used in suspension agents are wetting agents, preservatives, and UV-protective agents (Shi et al., 2008). When 650 μL Tween-80, 164.58 mg sodium citrate, and 308.12 mg sodium lignosulfonate were added to 65 mL *Bacillus amyloliquefaciens* fermentation broth, they controlled 84.78% of apple rot (Sun C. H et al., 2017). The addition of 0.5% folic acid, 0.5% tyrosine, and 1% riboflavin in yeast reduced the mortality of ultraviolet (UV) irradiation yeast (Lahlali et al., 2011).

Cereal crops are the most important food crops, and their yield accounts for about 90%. Cereal root rot can occur during the whole growth period and reduce yield by 20–30%, even 50% in severely affected plots (Tunali et al., 2008; Gupta et al., 2017; Li et al., 2019). The main pathogens are *Bipolaris sorokiniana*, *Fusarium graminearum*, and *Fusarium equiseti* (Kazan and Gardiner, 2018; Al-Sadi, 2021). Oat is one of the eight major grain crops of cereal crops. Oat is a one-year-old cereal crop and the field incidence of oat root rot diseases is 4–15%, and the main pathogens are *F. avenaceum, F. solani, F. graminearum, Gibberella moniliformis*, and *Gibbere*lla acuminata (Yang et al., 2021). Root rot is mainly controlled by biological agents in central and northern America and northern Italy (Parikh and Adesemoye, 2018; Colombo et al., 2019), as well as in crop-planting areas in China (Sun, 2019). Although the effects of biological control are remarkable, some problems such as single composition, uneven distributions, poor UV resistance, and resultant pollution have also been reported (O'Brien, 2017; Brodeur et al., 2018). Thus, it is necessary to develop microbial biocontrol agents with high activity, long action time, UV protection, and uniform dispersion. Therefore, the objectives of this study are to (1) obtain biocontrol bacteria with high control effect of pathogenic fungi, (2) select the best protective agents for antagonistic bacteria, and (3) verify the effectiveness of the biocontrol agent on oat.

## 2. Materials and methods

### 2.1. Test strains and culture medium

The eight bacteria and three pathogenic fungi were provided by the laboratory of grassland microbiology, Gansu Agricultural University, China (Tables 1, 2). The tested bacteria were cultured in a Luria–Bertani (LB) medium, and the pathogenic fungi were cultured in a potato dextrose agar (PDA) medium.

**Table 1:** The basic information of pathogenic fungi includes name, host, location and gene sequence number.

Number	Strains	Host plant	Disease site	Gene bank sequence
PB1	*Bipolaris sorokiniana*	wheat	root	MW494590
PB6	*Fusarium avenaceum*	highland barley	root	MW494595
PB7	*Fusarium equiseti*	oat	root	MW494596

**Table 2:** The host, characteristics and references of the test bacteria.

Code	Strains	Host plant	Nitrogenase activity(C_2_H_4_ nmol/(mL·h))	Amount of dissolved phosphorus(mg/L)	Secreting plant hormones(mg/L)	Source
GAU24	*Bacillus velezensis*	*Polygonum viviparum*	–	–	36.57	Zhang (2019); Liu (2016)
GAU39	*Bacillus xiamenensis*	*Allium fistulosum*	28.86	362.60	1.71	Zhang (2019); Jiang et al. (2018)
GAU68	*Bacillus mycoides*	*Kobresia myosuroides*	3193.07	67.15	66.21	Liu (2016)
GAU85	*Serratia plymuthica*	*Medicago sativa*	110.45	75.22	15.30	Sun G. Z et al., 2017
GAU86	*Bacillus pumilus*	*Triticum aestivum*	–	200.02	54.36	Liu et al. (2011)
GAU88	*Brevundimonas faecalis*	*Medicago sativa*	75.34	132.60	47.25	Zhang (2019); Han et al. (2013)
GAU89	*Bacillus subtilis*	*Trifolium pratense*	497.70	103.50	10.56	Sun et al. (2015)
GAU117	*Bacillus* sp.	*Triticum aestivum*	–	202.00	–	Feng et al. (2009)

### 2.2. Test reagents

The wetting agents consisted of Tween-20, Tween-80, and OP emulsifiers, purchased from Beijing Solarbio Technology Co. Ltd., China. The UV-protective agents were sodium alginate, sorbitol, and xanthan gum, ordered from Tianjin Tianchen Chemical Co. Ltd., China. The preservatives included methylparaben, ethylparaben, kaisong, and sodium citrate, purchased from Tianjin Guangfu Fine Chemical Co. Ltd., China. The protein peptone, yeast powder, agar, and glucose were purchased from Beijing Aobox Biotechnology Co. Ltd., China. The glucose and sodium chloride were purchased from Sinopharm Chemical Reagent Co. Ltd., China.

### 2.3. Screening bacteria and preparation of bacterial suspension

The antagonistic activity of the tested bacteria against pathogenic fungi was evaluated on PDA plates by the dual culture method. The tested bacteria were inoculated in a liquid LB medium at 25°C, 180 r/min for 24 h to achieve a 10^8^ cfu/mL concentration. Pathogenic fungi were grown on a PDA medium at 25°C for 5 d. The mycelial disk (5 mm) was placed at the center of the PDA medium, and bacterial suspension (100 uL) was spotted at 2 cm juxtaposed from the fungal disk four times with four replicates. The plates were incubated at 28°C for 3–7 days. The percentage of growth inhibition (I) was calculated by measuring the distance between the edges of the bacterium and fungal colonies by the following calculation (Sun G. Z et al., 2017):


$$I\left(\%\right)=[\left(C-T\right)/\left(C-C_{0}\right]\times 100\%$$

where I represents the inhibition rate, C indicates the colony radius of the fungi in control, T is the colony semidiameter of the fungi in the dual culture, and C_0_ means the radius of the test fungi agar disks.

To test the antifungal activity of the cell-free fermentation liquid (Sun G. Z et al., 2017), 2% (V/V) bacterial suspension was inoculated to the liquid LB medium and placed in a shaker at 28°C for 48 h at 180 r/min. The fermentation liquid was then centrifuged at 8000 r/min for 10 min and filtered by 0.22 μm filtration membranes. The media incorporating the filtrate at a volume fraction of 10% were inoculated with agar disks containing the tested fungi and sterile water (10% by volume, control) with three replicates and then incubated at 28°C for 3–7 days. The radius of mycelium growth of the fungi (mm) in both the treated (T) and control (C) petri dishes was measured in perpendicular directions until the fungi growth in the control dishes was almost complete. The percentage of growth inhibition (I) was calculated using the formula:


$$I\left(\%\right)=[\left(C-T\right)/\left(C-C_{0}\right]\times 100\%$$

where I represents the inhibition rate, C indicates the colony radius of the fungi in control, T is the colony semidiameter of the fungi in the dual culture, and C_0_ means the radius of the test fungi agar disks.

The bacterium combination was screened by the Oxford cup method (Sun G. Z et al., 2017). The Oxford cup method means that the growth of bacteria in the range of the bacteriostatic concentration around the Oxford cup is inhibited, forming a transparent bacteriostatic ring. In all, 100 μL of filtrate of one bacterium and 100 μL sterile water (control) were dropped into the Oxford cup (diameter 7 mm) at the center of the solid LB agar culture containing 2% of another bacterium. The plates were observed for inhibition zone after 24 h of incubation at 28°C, and the experiment was replicated thrice (Sun G. Z et al., 2017). The selected bacterium was inoculated in 50 mL liquid LB and shaken at 180 r/min, 28°C for 72 h. Then, the fermentation of each seed strain with 50 mL was mixed in a flask containing 500 mL of liquid LB medium and shaken at 180 r/min, 28°C for 72 h, and then put in a 4-degrees refrigerator spare.

### 2.4. Additive selection and microbial agents making

To select different additives, a single additive was added to a flask containing 50 mL of liquid LB medium and 5 mL of mixed bacteria (Table 3), using no additive one as control, with five replicates. Then, the sealed flasks were put at 180 r/min, 28°C for 40 h, and OD_600_ was determined by an ultraviolet spectrophotometer every 4 hours to draw the growth curve of the mixed bacteria.

**Table 3:** Experimental design of different chemical additive concentration.

Auxiliary type	Chemical additives	Concentrations/ (%)				
Wetting agents	Tween-20	0.10	0.50	1.00	1.50	2.00
	Tween-80	0.10	0.50	1.00	1.50	2.00
	OP emulsifier	0.05	0.10	0.50	1.00	2.50
UV-protectors	Sodium alginate	0.10	0.50	1.00	2.50	5.00
	Xanthan gum	0.10	0.50	1.00	2.50	5.00
	Sorbitol	0.05	0.10	0.20	0.50	1.00
Preservatives	Methylparaben	0.05	0.10	0.50	1.00	1.50
	Ethylparaben	0.05	0.10	0.50	1.00	1.50
	Kathon	0.05	0.15	0.25	0.50	1.00
	Sodium citrate	0.05	0.10	0.50	1.00	1.50

Based on the results of the single-additive experiment, the optimal wetting agent, UV-protection agent, preservative, and their concentration for the mixed bacteria were screened. The Box–Behnken design was used for the response surface analysis of the optimal additives and concentrations for the mixed bacteria growth (Barrera et al., 2019). The Box–Behnken central composite test was adopted using appropriate concentrations of different optimal additives as independent variables and the OD_600_ value of the mixed bacterial suspension as a response value. The quadratic regression was used to analyze the central composite test results to verify the fitting effect of the model, and the optimal combination of the concentration ratio of additives was determined.

To make the fungi inhibitor, the additives were added into a liquid LB medium containing mixed bacteria according to their best combination and placed at 180 r/min at 28°C for 72 h before being stored at room temperature.

To detect the quality of the inhibitor, two sample were taken every 15 d until 90 days (Wang et al., 2021). One sample was diluted to 10^8^ cfu/mL using sterile water, taken at 20 μL and coated on a solid LB medium with five replicates, and then incubated at 28°C for 48 h to count the number of living bacteria and contaminating microorganisms. The other sample was used to determine the pH. The calculation formula used is as follows:


BN(cfu/mL)=AN×DM×BV/(SS×SA)

MR(%)=IC/(IC+LC)×100%

where BN represents the number of colonies, AN is the average bacteria count, DM means the dilution multiple, BV denotes the base liquid volume, SS indicates the sample volume, SA is the pipetting volume, MR means the mixed bacteria rate, IC indicates the microbial contaminant, and LC is the effective viable count.

### 2.5. Biocontrol efficacy evaluation of microbial inhibitor

To test the effectiveness of the fungi inhibitor, a pot experiment was conducted. Oat seeds were disinfected with 1% NaClO for 1 min, and 10 were seeded in each plastic pot (80 mm × 45 mm × 205 mm) containing farmland soils. The pots were put in an incubator (25°C/18°C, 16 h/ 8 h) for 1 w before being thinned to seven plants. The oat variety was Longyan 3, which was provided by the College of Grassland Science, Gansu Agricultural University, China.

The pathogenic fungi PB1 (*Bipolaris sorokiniana*), PB6 (*Fusarium avenaceum*), and PB7 (*Fusarium equiseti*) were scraped onto PDA plates and incubated at 25°C for 7 days; then, spores of the pathogenic fungi were dispersed into a 6 g/L carboxymethyl cellulose solution to produce a 10^7^ cfu/mL spore suspension.

Two weeks after thinning, the 5 mL spore suspension and sterile water (control) were inoculated (Table 4) by the perfusion method (Zheng et al., 2019). A five-milliliter microbial inhibitor (MA) was injected into each pot 7 days after inoculation. The root rot disease symptoms were recorded 21 days after the pathogenic fungi inoculation.

**Table 4:** Pot-experimental design.

Code	Control	Treatment
CK	PB (sterile water)	PB + MA (Microbial inhibitor)
T1	PB1 (*Bipolaris sorokiniana*)	PB1 + MA (*B. sorokiniana* + MA)
T6	PB6 (*Fusarium avenaceum*)	PB6 + MA (*F. avenaceum* + MA)
T7	PB7 (*Fusarium equiseti*)	PB7 + MA (*F. equiseti* + MA)

Six oat plants were selected for each treatment, and plant height, fresh biomass, and root length were determined. Disease was ranked according to Table 5, and disease incidence was calculated as per the following equation:


I(%)=TD/TI×100%

DI=[∑(TL×RV)/(TI×ML)]×100

CE(%)=(CI−TI)/CI×100%

where I means incidence, TD represents the total number of diseased plants, TI is the total number of plants investigated, DI denotes the disease index, TL signifies the total number of diseased plants at all levels, RV means the representative value, ML denotes the maximum disease level representation, CE represents the control effect, CI is the control disease index, and TI signifies treatment of the disease index.

**Table 5:** Classification standard of root rot diseases.

Rank	Occurring degree	Representative value
1	disease-free	0
2	Disease spot accounts for 1% ~ 5% of root surface area	1
3	Disease spot accounts for 6% ~ 10% of root surface area	2
4	Disease spot accounts for 11% ~ 20% of root surface area	3
5	Disease spot accounts for 21% ~ 40% of root surface area	4
6	Disease spot accounts for 41% ~ 60% of root surface area	5
7	Disease spot accounts for 61% ~ 80% of root surface area	6
8	Disease spot accounts for more than 80% of root surface area	7

### 2.6. Data analysis

The data of the inhibition of mycelial growth of the tested bacteria against the pathogenic fungi, the Box–Behnken central composite experiment, and the control effect of the microbial inhibitor on oat root diseases were analyzed using analysis of variance (ANOVA) for individual parameters on the basis of mean values to find out the significance at a 5% level. The standard errors of the mean and ANOVA statistics were calculated using SPSS 22.0. Design Expert was used for experimental design response surface optimization analysis, and Origin software was used for plotting.

## 3. Results

### 3.1. Effect of tested bacterium on the growth of pathogenic fungi

#### 3.1.1. *In vitro* antifungal activity of tested bacterium and liquid cell-free fermentation

**Figure 1:**
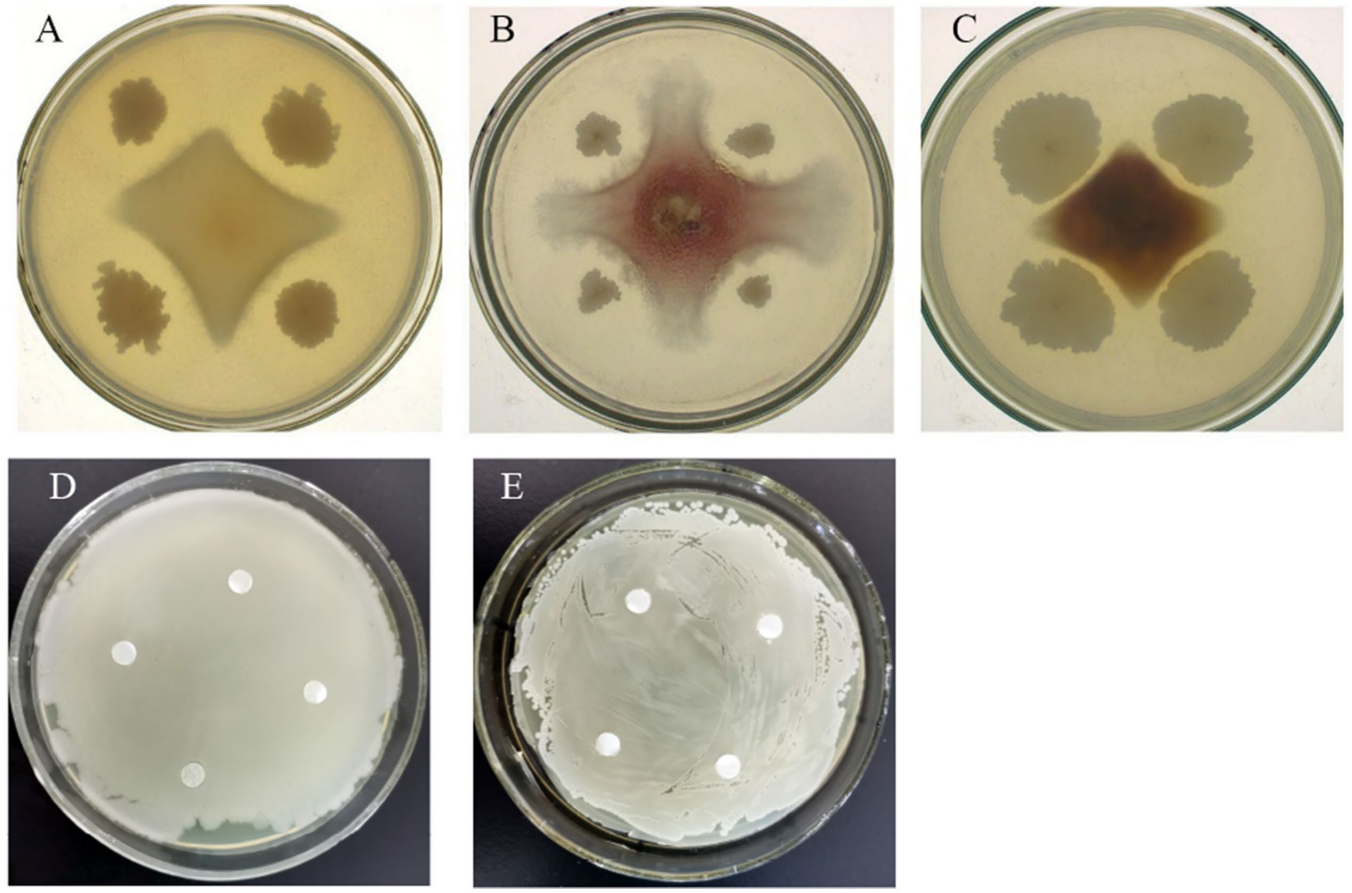
Confrontation between two strains (part). **A** represented the inhibitory effect of GAU88 (*Brevundimonas faecalis*) on *Bipolaris sorokiniana*; **B** was the inhibitory effect of GAU39 (*Bacillus xiamenensis*) on *Fusarium avenaceum*; **C** meant the inhibitory effect of GAU24 (*Bacillus velezensis*) on *Fusarium equiseti*; **D** and **E** denoted the co-growth effect of GAU24 (*B. velezensis*) on GAU88 (*B. faecalis*).

Different bacteria had different inhibition effects on pathogenic fungi growth (Figure 1). After 7-day cultivation, the antifungal activity of the tested bacteria varied from 50.00 to 83.29%, whereas liquid cell-free fermentation showed a 8.35–39.90% inhibition rate (Table 6). Moreover, GAU88 had the highest antifungal activity on *Bipolaris sorokiniana* (83.29%) and *Fusarium avenaceum* (74.56%), while GAU68 was most effective (75.30%) on *Fusarium equiseti*. Among the eight bacteria, GAU24, GAU68, and GAU88 had better inhibitory effects on the three pathogenic fungi, while liquid cell-free fermentation of GAU24 and GAU88 also gave better performance.

**Table 6:** Inhibition of mycelial growth of the tested bacteria against pathogenic fungi.

Strain n_O_.	PB1	PB6	PB7	PB1	PB6	PB7
		m			n	
GAU24	64.15 ± 2.05c	50.00 ± 2.20d	63.48 ± 0.95 cd	34.85 ± 1.19b	25.05 ± 1.39a	26.47 ± 0.93b
GAU39	0.00 ± 0.00d	56.12 ± 2.25c	67.78 ± 1.41bc	0.00 ± 0.00c	15.13 ± 1.28b	8.35 ± 1.06d
GAU68	0.00 ± 0.00d	62.24 ± 2.01b	75.30 ± 1.18a	0.00 ± 0.00c	8.37 ± 0.49c	8.53 ± 0.80d
GAU85	0.00 ± 0.00d	0.00 ± 0.00e	62.41 ± 0.94d	0.00 ± 0.00c	0.00 ± 0.00d	22.51 ± 1.01c
GAU86	67.47 ± 1.22b	0.00 ± 0.00e	0.00 ± 0.00e	32.64 ± 1.15b	0.00 ± 0.00d	0.00 ± 0.00e
GAU88	83.29 ± 2.05a	74.56 ± 1.09a	64.55 ± 0.61bcd	39.90 ± 1.85a	23.51 ± 0.67a	31.37 ± 1.59a
GAU89	0.00 ± 0.00d	64.29 ± 1.77b	68.85 ± 1.70b	0.00 ± 0.00c	18.27 ± 1.22b	0.00 ± 0.00e
GAU117	0.00 ± 0.00d	54.08 ± 2.01c	60.24 ± 1.19d	0.00 ± 0.00c	0.00 ± 0.00d	0.00 ± 0.00e

PB1 was Bipolaris sorokiniana; PB6 meant Fusarium avenaceum; PB7 denoted Fusarium equiseti; GAU24 signified Bacillus velezensis, GAU39 was Bacillus xiamenensis, GAU68 indicated Bacillus mycoides Serratia, GAU85 signified Serratia plymuthica, GAU86 meant Bacillus pumilus, GAU88 was Brevundimonas faecalis, GAU89 was Bacillus subtilis, GAU117 represented Bacillus sp. The letter m antifungal activity of tested bacterium. The n antifungal activity of cell-free fermentation liquid. Values in the table are mean ± SE. Different letters within the same column indicate significant difference at P < 0.05 level by Duncan’s test.

#### 3.1.2. Screening of bacterium combinations

Bacteriostatic ring was not observed after one day of bacteria in the Oxford cup, indicating a good growth. According to the result from Table 6, GAU24 and GAU88 were finally selected and tested for antagonism by the Oxford cup method, which showed no antagonism; thus, they could coexist (Figures 1D,E).

### 3.2. Screening of auxiliary agents for antifungal bacteria

#### 3.2.1. Screening of the wetting agents

**Figure 2:**
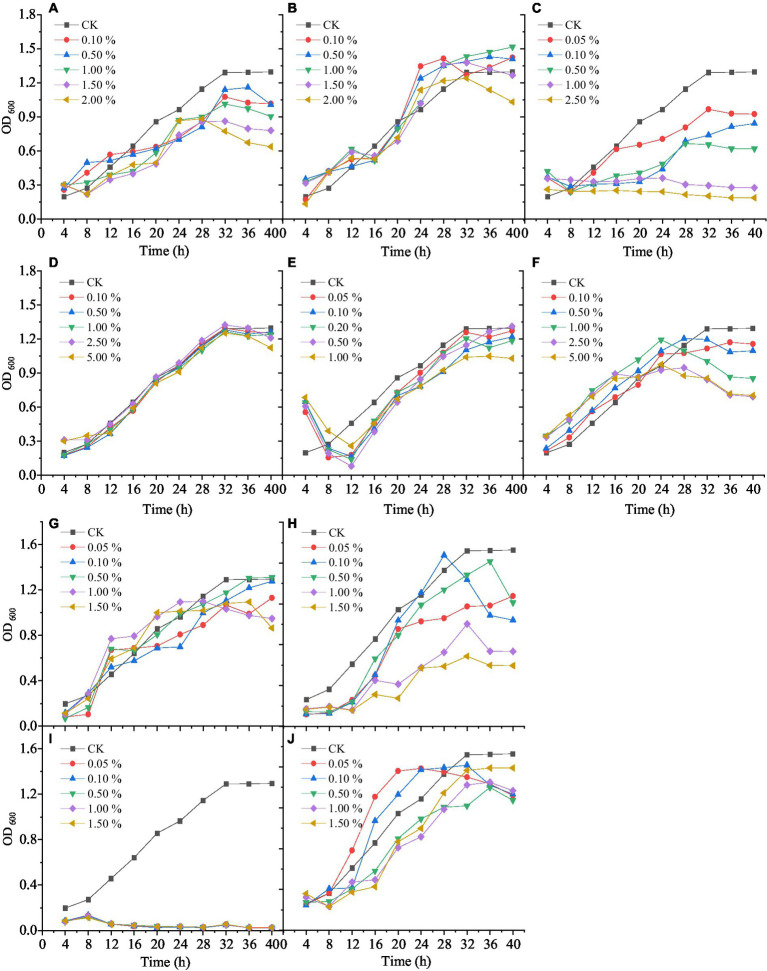
Effect of different types and concentrations of auxiliary agents on mixed biocontrol strains growth. **A:** Tween-20; **B:** Tween-80; **C:** OP emulsifier; **D:** sodium alginate; **E:** sorbitol; **F:** xanthan gum; **G:** methyl paraben; **H:** ethyl paraben; **I:** kathon; **J:** sodium citrate.

Wetting agents can increase the activity of mixed biocontrol bacteria (MBB). Different concentrations of Tween-20, Tween-80, and OP emulsifier had different influence on the growth activity of MBB (Figures 2A–C). MBB grew rapidly and then slowly with the prolonging culture. Tween-80 had little influence on MBB growth, while Tween-20 and OP emulsifier had strong inhibitory effects on the growth of MBB. After 12 h culture, the OD_600_ value of the OP emulsifier-treated MBB was 48.42% (*p*<0.05) lower than the control. After 24 h culture, the OD_600_ value of Tween-80-treated MBB was 5.80% higher than the control, and when its mass fraction was 1.00%, the OD_600_ value of MBB reached 1.471 (10.91% greater than the control, *p* < 0.05) after 32 h culture. Therefore, Tween-80 was most suitable as a wetting agent for MBB with 1.00% mass fraction.

#### 3.2.2. Screening of ultraviolet protective agents

Ultraviolet protection agents can reduce the ultraviolet damage of MBB and improve their activity. Different concentrations of sodium alginate, sorbitol, and xanthan gum had different effects on the growth activity of MBB (Figures 2D–F). With the increase of culture time, the growth of MBB increased and then decreased after adding sodium alginate, sorbitol, and xanthan gum. After 28 h culture, xanthan gum inhibited MBB growth, as indicated by the much lower OD_600_ value than that of the control. When the mass fraction of sorbitol was 0.50%, the MBB’s OD_600_ value was higher than that of the control (1.411 vs. 1.296, *p* < 0.05) after 40 h culture. Thus, sorbitol was selected as a UV-protective agent for MBB with 0.50% mass fraction.

#### 3.2.3. Screening of preservatives

Preservatives can reduce bacterial contamination and prolong the storage time of MBB. Adding different concentrations of methylparaben, ethylparaben, Kathon, and sodium citrate had different effects on the growth of MBB (Figures 2G–J). With the increase of culture time, compared with the control, Kathon and ethylparaben inhibited MBB, and Kathon was the most effective inhibitor. However, low concentrations of methylparaben and sodium citrate promoted MBB growth. At 0.50% mass fraction of methylparaben, the OD_600_ value of MBB was 8.82% greater than that of the control (*p*<0.05). Therefore, methylparaben was used as a preservative for MBB with 0.50% mass fraction.

### 3.3. The ratio optimization of additives

Based on a single-factor test, a 3 × 3 response surface analysis experiment was conducted for the mixed biocontrol agents (Table 7). According to the Box–Behnken central composite experiment (Table 8), 750 μL Tween-80, 300 mg sorbitol, and 300 mg methylparaben were added into the mixed biocontrol bacterial suspension, and the highest OD_600_ value was 1.51. Data regression analysis was performed using the RSA program, and the regression equation models between the OD_600_ value and three influencing factors were as following:


$$Y=1.45+Y_{1}+Y_{2}+Y_{3}$$

$$Y_{1}=0.071A+0.061B+0.083C$$

$$Y_{2}=-0.050\mathrm{AB}+0.14\mathrm{AC}-0.058\mathrm{BC}$$

$$Y_{3}=-0.054A^{2}-0.094B^{2}-0.19C^{2}$$

where A, B, and C were Tween-80, sorbitol, and methylparaben, respectively.

**Table 7:** The test factor levels of the Box–Behnken.

Code	Factor		Factor levels	
		-1	0	1
A	Tween-80 /%	0.50	1.00	1.50
B	Sorbitol /%	0.20	0.50	1.00
C	Methylparaben /%	0.10	0.50	1.00

**Table 8:** Box–Behnken central composite experiment.

Number	Factors			
	A: Tween-80/μL	B: Sorbitol/mg	C: Methylparaben /mg	Y: OD_600_ value
1	550	500	100	1.21 ± 0.01f
2	550	500	300	1.25 ± 0.01e
3	350	500	200	1.35 ± 0.03d
4	550	300	200	1.48 ± 0.02b
5	350	300	300	1.08 ± 0.03i
6	350	300	100	1.18 ± 0.02 g
7	550	300	200	1.49 ± 0.01b
8	550	300	200	1.37 ± 0.03d
9	750	300	300	1.51 ± 0.01a
10	550	300	200	1.49 ± 0.01b
11	750	100	200	1.36 ± 0.02d
12	750	500	200	1.38 ± 0.02d
13	550	300	200	1.44 ± 0.03c
14	550	100	300	1.24 ± 0.01e
15	750	300	100	1.06 ± 0.03i
16	350	100	200	1.13 ± 0.03 h
17	550	100	100	0.97 ± 0.04j

Different letters within the same column indicate significant difference at *p* < 0.05 level by Duncan’s test.

The regression model was significant (*p* < 0.0001, Table 9). Three different types of additives had significant effects on the growth of MBB. The equation’s lack fit was 0.9748 > 0.05, indicating a stable model with accurate predicted value. The coefficient of determination R^2^ = 0.9759, showing a good fitting degree. The influence of each additive on the OD_600_ value of MBB activity could be judged by the *F* value. The greater the F value, the greater the influence.

**Table 9:** Variance analysis of the regression equation.

Source	Sum of squares	*DF*	Mean square	*F*	*P*(Prob) > F	*P*(Prob) > F
Model	0.45	9	0.050	31.43	<0.0001	**
A	0.041	1	0.041	25.74	0.0018	**
B	0.030	1	0.030	19.02	0.0042	**
C	0.054	1	0.054	34.51	0.0008	**
AB	0.010	1	0.010	6.34	0.0496	*
AC	0.076	1	0.076	47.93	0.0003	**
BC	0.013	1	0.013	8.38	0.0284	*
A^2^	0.013	1	0.013	7.93	0.0415	*
B^2^	0.038	1	0.038	23.83	0.0022	**
C^2^	0.16	1	0.16	98.37	<0.0001	**
Residual	0.011	7	0.001			
Lack of fit	0.0005	3	0.00017	0.067	0.9748	ns
Pure error Lack of fit	0.011	4	1.83			
Cor total	0.46	16				
Adj R^2^	0.9759					

DF represented degree of freedom; **represented significant at *p* < 0.01; *represented significant at *p* < 0.05；ns represented not significant at *p* > 0.05.

**Figure 3:**
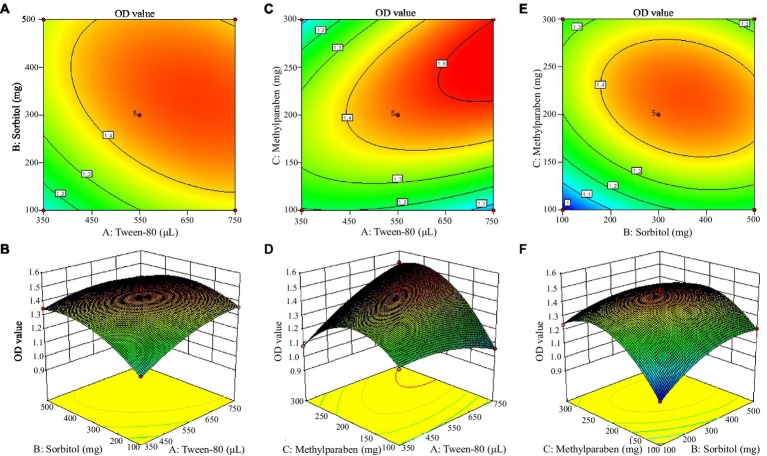
Contour plot (up) and surface (down) of mutual-influence for the selected adjuvant. Letter in the figure, **A** was contour plot and **B** meant surface of mutual-influence for the tween-80 and sorbitol; **C** denoted contour plot and **D** indicated surface of mutual-influence for the tween-80 and methylparaben; **E** was contour plot and **F** meant surface of mutual-influence for the sorbitol and methylparaben.

The response surface is a three-dimensional surface figure composed of response values and tested factors. Figure 3 shows that Tween-80 and methylparaben had the strongest interaction. The Design-Expert 10 software gave the optimal combination formula, i.e., 14.96 mL/L Tween-80, 5.60 g/L sorbitol, and 5.12 g/L methylparaben, under which the OD_600_ value of MBB was 1.53, very close to the result shown in Table 8 (1.51 OD_600_ value), thus indicating an accurate and reliable result of the response surface method.

### 3.4. Quality inspection of microbial inhibitor

#### 3.4.1. Living bacteria count of microbial inhibitor

**Figure 4:**
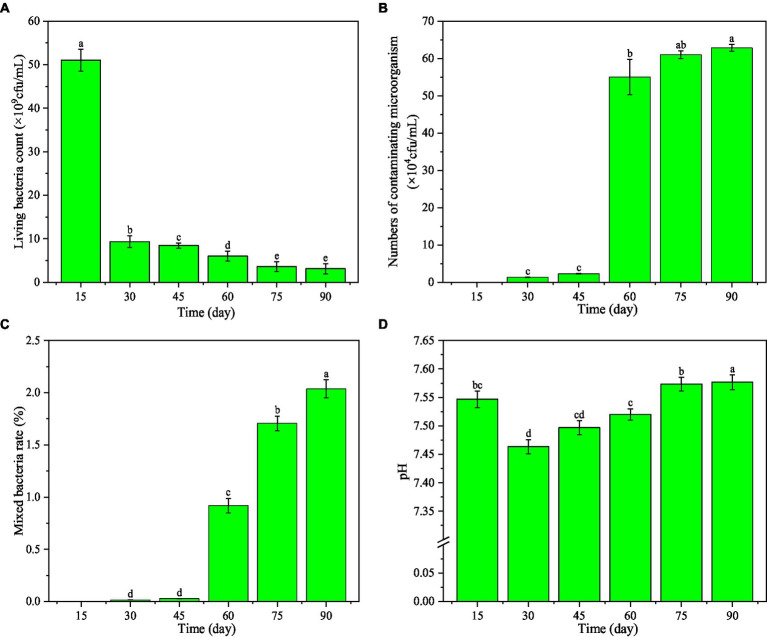
Effect of storage time on the main indexes of microbial inhibitor. Different letters within different storage time indicate significant difference at *p* < 0.05 level by Duncan’s test.

As the storage time of the microbial inhibitor prolonged, the living bacteria count decreased (Figure 4A). It was 5.10 × 10^9^ cfu/mL on the 15th day, which was 5.46 times that on the 30th day and 16.45 times that on the 90th day. The living bacteria count was 3.10 × 10^9^ cfu/mL on the 90th day, still much greater than that required by China National Standard GB 20287–2006 (1.00 × 10^8^ cfu/mL).

#### 3.4.2. The number and rate of contaminating microorganisms in microbial inhibitor

With the increase of the storage time of the microbial inhibitor, the number of contaminating microorganisms was 6.28 × 10^5^ cfu/mL on the 90th day(Figure 4B), which was 1.14 times that on the 60th day (*p* < 0.05). However, the contaminating microorganism count was far less than 10^4^ cfu/mL (GB 20287–2006). Moreover, the mixed bacteria rate on the 90th day was 2.22 times that on the 60th day and 1.19 times that on the 75th day (Figure 4C), but it was still much lower than required by GB 20287–2006 (3.0 × 10^6^ cfu/mL).

#### 3.4.3. pH value of microbial inhibitor

The pH of the microbial inhibitor increased initially and then decreased with the increasing storage time (Figure 4D). It was 7.55 on the 15th day, then declined to 7.46 on the 30th day, and rose up to 7.58 on the 90th day. This range is in accordance with GB 20287–2006.

### 3.5. Control effect of microbial inhibitor on oat root diseases and growth of oat

**Figure 5:**
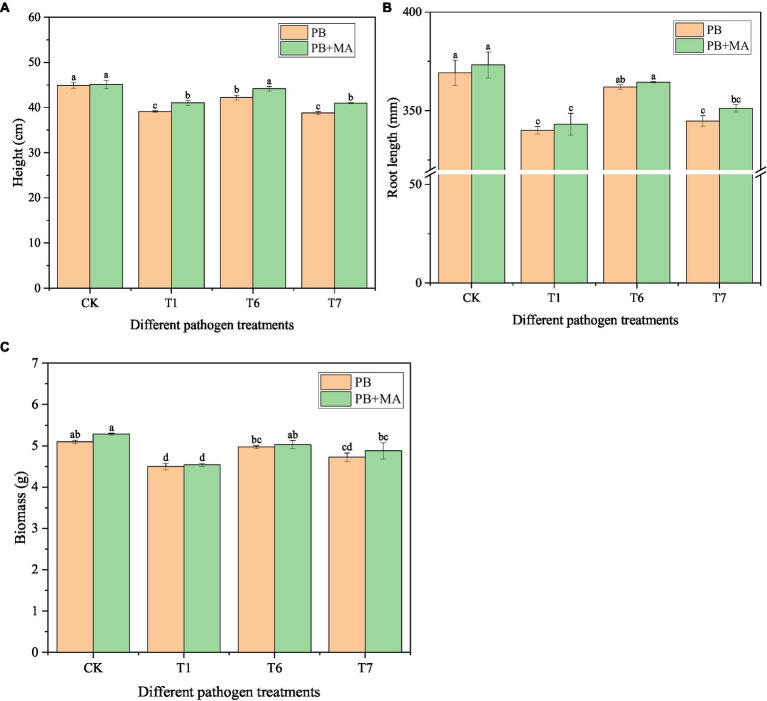
Effect of microbial inhibitor on oat seedling stage growth. PB in CK and in T treatment represent sterile water and pathogenic fungi respectively; MA was microbial inhibitor. Different letters within the treatment indicate significant difference at *p* < 0.05 level by Duncan’s test.

The incidence of three pathogenic fungi on oat roots was 34.71% (T1), 30.02% (T7), and 25.84% (T6) (Table 10). The highest disease index was 27.68 from T1. The highest control effect of the microbial inhibitor was observed in T6 (68.44%), followed by T7 (52.96%) and T1 (48.70%). Accordingly, the best plant growth was obtained in the T6 treatment with a taller plant (*p* < 0.05), longer root length, and greater biomass (Figure 5).

**Table 10:** Control effect of microbial inhibitor on oat root diseases.

Code	Incidence(%)	Disease index	Control effect(%)
T1 (*Bipolaris sorokiniana*)	34.71 ± 1.94a	27.68 ± 0.55a	48.70 ± 0.57c
T6 (*Fusarium avenaceum*)	25.84 ± 0.35c	16.98 ± 0.38c	68.44 ± 0.47a
T7 (*Fusarium equiseti*)	30.02 ± 0.75b	23.28 ± 0.61b	52.96 ± 1.15b

Different letters within the same column indicate significant difference at *p* < 0.05 level by Duncan’s test.

## 4. Discussion

Some biocontrol bacteria produce resistant substances against pathogens and play important roles in disease biocontrol (Compant et al., 2005; Kaewkham et al., 2016). *Bacillus velezensis*, *Bacillus xiamenensis*, *Bacillus mycoides*, and *Brevundimonas faecalis* significantly inhibited the growth of the three pathogens in this study. Similar results were also reported by Ait-Kaki et al. (2014), who obtained 60.00 and 61.00% inhibitory rates of *B. velezensis* isolated from *Calendula officinalis* against *F. oxysporum* and *Botrytis cinerea*, respectively. Moreover, 66.00 and 56.00% inhibitory rates of *B. velezensis* isolated from *Cucumis sativus* against *F. oxysporum* were obtained by Liu et al. (2017). *Streptomyces* sp., *Saccharothrix* sp., and *Nocardpsis* sp. isolated from the rhizosphere of *Solanum tuberosum* were shown to significantly inhibit the mycelial growth of *Phytophthora infestans* with a 35.02–79.20% inhibitory rate (Feng et al., 2022). Bacteria isolated from rice leaves have antagonistic effects on *Rhizoctonia solani* and inhibit disease spot extension *in vitro* (Shrestha et al., 2016). Inhibitory rates as high as 73.82, 66.81, and 85.71% of biocontrol bacteria on *Sclerotinia sclerotiorum*, *F. oxysporum,* and *R. solani*, respectively, were reported (Sun G. Z et al., 2017). In this study, cell-free fermentation liquid of biocontrol bacteria also inhibited the growth of the pathogenic fungi. The fermentation broth of GAU88 had inhibitory effects on *B. sorokiniana* (PB1) and *F. avenaceum* (PB6), with inhibitory rates of 39.90 and 31.37%, respectively; even higher inhibitory rates (67.00 and 54.00%) of the cell-free fermentation liquid of *B. velezensis* isolated from *Cucumis sativus* against *F. oxysporum* have been observed (Liu et al., 2017). *Bacillus* and *Brevundimonas* may produce antimicrobial proteins (Kim et al., 2017) and lipopeptide antibiotics (Peypoux et al., 1978, 1999; Vanittanakom et al., 1986) and secrete growth hormones (Meng et al., 2016; Yaashikaa et al., 2020), pyoverdine, and NH_3_ (Meng et al., 2016). It can be seen that biocontrol bacteria have a good inhibitory effect on the growth of pathogenic fungi. In order to make biocontrol bacteria play a better role, it is a good choice to add preservatives, protective agents, and wetting agents to them.

Biocontrol bacteria are viable microorganisms and sensitive to UV, extreme temperature, light, and other environmental factors (Liu and Xing, 2016). To reduce these impacts and prolong the shelf life of microbial preparations, it is necessary to add appropriate protective additives (Arora and Mishra, 2016). At present, most auxiliary additives are chemically synthesized, and so the compatibility of the additives with living microorganisms should be fully considered. The addition of Tween-80 to the culture medium of *Streptomyces padanus* was demonstrated to enhance the inhibitory effect of *S. padanus* against cucumber downy mildew (Fan et al., 2019). Furthermore, the addition of 0.1% Tween-80 to laboratory growth media increased the growth rate of planktonic *Staphylococcus aureus* batch cultures, and it also increased the total biomass when *S. aureus* was grown as biofilms (Nielsen et al., 2016). Studies have shown that sorbitol may act as an antioxidant to scavenge reactive oxygen species and precisely regulate the balance of reactive oxygen species under the synergistic effect of antioxidant enzyme systems (SOD, POD, CAT) (Smirnoff and Cumbes, 1989). Parabens are a class of compounds primarily used as antimicrobial preservatives in pharmaceutical products, cosmetics, and foodstuffs (Nguyen et al., 2021). It was shown that in a control of grape downy mildew, a combination of corn steep liquor, lignosulfonate, and polyethylene glycol in *Lysobacter capsici* formula increased the survival rate of *L. capsici* in the field and reduced the occurrence of disease by 71%; in addition, the authors also admitted that the required quantities limited the usefulness of that particular formulation (Segarra et al., 2015). In this study, adding 1.00% Tween-80, 0.50% sorbitol, and 0.50% methylparaben to 50 mL of mixed fermentation had no inhibitory effects, indicating that the developed biocontrol bacteria had good compatibility with the additives. During the growth of biocontrol bacteria, their viable counts decrease due to the decline of nutrients and oxygen and the production of lactic acid, causing fluctuation of the pH (Wong et al., 2019). The microbial inhibitor developed in this study was in compliance with the China National Standards of Agricultural Microbial Agents (GB 20287–2006) in terms of living bacteria counts, contaminating microorganism counts, undesirable mixed bacteria rate, and pH.

To test the effectiveness of the developed microbial inhibitor, the pathogenic fungi and microbial inhibitor were inoculated on oat, and the results showed as a control effect as high as 68.44%, much higher than that of *Bacillus cereus* on tobacco bacterial wilt (31.43%, Wu et al., 2020). *Pseudomonas brassicacearum*, *Pseudomonas putida*, *Paenibacillus peoriae*, and *Bacillus licheniformis* isolated from potato have been shown to reduce potato disease occurrence by 27–55% (Bahmani et al., 2021). Plant growth-promoting rhizo-bacteria may interact with plants directly by increasing the availability of essential nutrients (nitrogen, phosphorus, and iron), production and regulation of compounds involved in plant growth (phytohormones), and stress hormonal status (ethylene levels by ACC-deaminase). They may also indirectly affect plants by protecting them against diseases through competition with pathogens for highly limited nutrients, biocontrol of pathogens through the production of aseptic-activity compounds, synthesis of fungal cell-wall-lysing enzymes, and the induction of systemic responses in host plants (Olenska et al., 2020). In this study, although the microbial inhibitor showed good control effects on the different pathogenic fungi in the pot experiments, their effects in fields need further study.

## 5. Conclusion

In conclusion, this study comprehensively analyzed the additive screening and formula optimization of a microbial inhibitor with disease prevention and growth promotion effects on *Avena sativa*. *B. velezensis* GAU24 and *B. faecalis* GAU88 had good inhibitory effects on *B. sorokiniana*, *F. avenaceum*, and *F. equiseti* and could be used to make the biocontrol agent. Their optimal additives consisted of 1.00% wetting agent Tween-80, 0.50% UV-protective agent sorbitol, and 0.50% preservative methylparaben, and the optimal combination formula was 14.96 mL/L Tween-80, 5.6 g/L sorbitol, and 5.12 g/L methylparaben. When used on oat, it could control 48.70–68.44% of root rot. In addition, the developed microbial inoculants have disease prevention and growth-promoting effects on plants. This study provides a suitable environment for the protection of mixed biocontrol bacteria and lays a foundation for the prevention and control of oat diseases, the promotion of growth, and the improvement of quality.

## Data availability statement

The original contributions presented in the study are included in the article/supplementary material, further inquiries can be directed to the corresponding authors.

## Author contributions

JZ, TY, and GZ designed the experiments and contributed to the writing and revision of the manuscript. JZ performed the experiments, being assisted by WG and YG, analyzed the data, and wrote the manuscript. All authors contributed to the article and approved the submitted version.

## Funding

This work was supported by China Agriculture Research System (CARS-07-C-1) and Excellent Doctoral Program Foundation of Gansu Province (22JR5RA841).

## Conflict of interest

The authors declare that the research was conducted in the absence of any commercial or financial relationships that could be construed as a potential conflict of interest.

## Publisher’s note

All claims expressed in this article are solely those of the authors and do not necessarily represent those of their affiliated organizations, or those of the publisher, the editors and the reviewers. Any product that may be evaluated in this article, or claim that may be made by its manufacturer, is not guaranteed or endorsed by the publisher.
